# PicXAA-R: Efficient structural alignment of multiple RNA sequences using a greedy approach

**DOI:** 10.1186/1471-2105-12-S1-S38

**Published:** 2011-02-15

**Authors:** Sayed Mohammad Ebrahim Sahraeian, Byung-Jun Yoon

**Affiliations:** 1 Department of Electrical and Computer Engineering, Texas A&M University, College Station, TX 77843, USA

## Abstract

**Background:**

Accurate and efficient structural alignment of non-coding RNAs (ncRNAs) has grasped more and more attentions as recent studies unveiled the significance of ncRNAs in living organisms. While the Sankoff style structural alignment algorithms cannot efficiently serve for multiple sequences, mostly progressive schemes are used to reduce the complexity. However, this idea tends to propagate the early stage errors throughout the entire process, thereby degrading the quality of the final alignment. For multiple protein sequence alignment, we have recently proposed PicXAA which constructs an accurate alignment in a non-progressive fashion.

**Results:**

Here, we propose PicXAA-R as an extension to PicXAA for greedy structural alignment of ncRNAs. PicXAA-R efficiently grasps both folding information within each sequence and local similarities between sequences. It uses a set of probabilistic consistency transformations to improve the posterior base-pairing and base alignment probabilities using the information of all sequences in the alignment. Using a graph-based scheme, we greedily build up the structural alignment from sequence regions with high base-pairing and base alignment probabilities.

**Conclusions:**

Several experiments on datasets with different characteristics confirm that PicXAA-R is one of the fastest algorithms for structural alignment of multiple RNAs and it consistently yields accurate alignment results, especially for datasets with locally similar sequences. PicXAA-R source code is freely available at: http://www.ece.tamu.edu/~bjyoon/picxaa/.

## Background

Increasing number of newly discovered non-coding RNAs (ncRNAs) with huge functional variety has revealed the substantial role that RNAs play in living organisms [1-3]. The function of ncRNAs is largely ascribed to their folding structure, which is often better conserved than their primary sequence. Therefore, it is important to consider this structural aspect in the comparative analysis of RNAs, and an accurate structural alignment algorithm can be helpful in decoding the function of ncRNAs and discovering novel ncRNA candidates.

To accurately align RNA sequences, one should take their secondary structure similarities into account, in addition to their sequence homologies. Simultaneous inference of both the consensus secondary structure and the alignment of RNA sequences is a computationally demanding task. Sankoff [4] proposed an algorithm for structural alignment of a set of unaligned RNA sequences. However, the high complexity of O(*L*^3^*^N^*) in time and O(*L*^2^*^N^*) in memory for *N* sequences of length *L* makes this algorithm impractical even for a small number of sequences. Hence, several studies have proposed various approximations to the Sankoff algorithm [5-19]. Algorithms such as Foldalign [5-7], Dynalign [8,9], and Stemloc [10] employ several heuristics to impose constraints on the size or shape of substructures, thereby, reducing the search space. Murlet [12], RAF [13], PARTS [14], STRAL [15], LocARNA [16], CentroidAlign [17], and PMcomp [18] exploit probabilistic approaches by implementing base-pairing probabilities in a restricted Sankoff-style framework or employing the Needleman-Wunsch algorithm with structural scores. Although these variants of Sankoff’s algorithm significantly reduce the time and memory complexities, they still cannot directly find the structural alignment of multiple sequences. Instead, these algorithms build up the multiple sequence alignment (MSA) by progressively combining pairwise structural alignments along a guide tree.

In addition to these Sankoff-style algorithms, several studies have recently investigated fast techniques to find the common structure of long RNA sequences. For example, MXSCARNA [20] progressively computes the pairwise structural alignment of a pair of stem candidates obtained from the base-pairing probability matrices. R-Coffee [21,22] uses a library of input alignments to progressively compute the alignment by incorporating secondary structure information. LARA [23] and MARNA [24] employ two different heuristic approaches to compute all pairwise structure alignments and pass this information, as a primary library, to T-COFFEE [25], a progressive alignment technique. MAFFT-xinsi [26] uses a four-way consistency objective function to progressively build a structural alignment by combining pairwise alignments predicted by an external program.

Despite its computational efficiency, the progressive structural alignment approach tends to propagate the errors made in the early stages throughout the entire process, which may significantly degrade the quality of the final alignment. Even with the incorporation of additional heuristics, such as iterative refinement and consistency transformation, the fundamental shortcoming of progressive technique remains. A number of non-progressive structural alignment schemes have been proposed to address this problem [27-29].

RNASampler [27] predicts the common structure of multiple RNA sequences by probabilistically sampling aligned stems based on the stem conservation score. MASTER [28], another sampling approach, iteratively improves both sequence alignment and structure prediction by making small local changes using simulated annealing. Stemloc-AMA [29] employs sequence annealing to construct the multiple RNA alignment using the base alignment probabilities estimated by the Sankoff algorithm with structural considerations.

Recently, several studies have highlighted the effectiveness of the Maximum Expected Accuracy (MEA) approach for aligning biological sequences [30-36] and for predicting the consensus secondary structure of RNAs [12,17,20,29,37-39]. MEA tries to maximize the expected number of correctly aligned bases. This is especially useful for handling sequence analysis problems when the probability of the optimal alignment is low.

In this paper, we introduce PicXAA-R (**p**robabilist**ic** ma**x**imum **a**ccuracy **a**lignment of **R**NA sequences), a novel non-progressive algorithm that efficiently finds the maximum expected accuracy structural alignment of multiple RNA sequences. PicXAA-R greedily builds up the structural alignment from sequence regions with high local similarities and high base-pairing probabilities. To simultaneously consider both the local similarities among sequences and their conserved secondary structural information, we incorporate three types of probabilistic consistency transformations. These transformations modify both the inter-sequence pairwise base alignment probabilities and the intra-sequence base-pairing probabilities using the information from other sequences in the alignment. For a fast and accurate construction of the alignment, we propose an efficient two-step graph-based alignment scheme. In the first step, we greedily insert the most probable alignments of base-pairs with high base-pairing probability. In this way, we build up the skeleton of the alignment using the structure information of the RNA sequences. Next, we successively insert the most probable pairwise base alignments into the multiple structural alignment, as in PicXAA [34], a multiple protein sequence alignment algorithm that we have recently proposed. This step can effectively grasp the local sequence similarities among the RNAs. Finally, we use a discriminative refinement step to improve the overall alignment quality in sequence regions with low alignment probability. Extensive experiments on several local alignment benchmarks clearly show that PicXAA-R is one of the fastest algorithms for structural alignment of multiple RNAs and it consistently yields accurate results in comparison with several well-known structural RNA alignment algorithms.

## Methods

PicXAA-R extends the idea of PicXAA, the multiple sequence alignment algorithm that maximizes the expected number of correctly aligned bases, to the structural alignment of RNA sequences. PicXAA-R uses a greedy approach that builds up the alignment from sequence regions with high local similarities and high base-pairing probabilities. Thus, it avoids the propagation of early stage alignment errors, usually observed in progressive techniques. The algorithm employs a probabilistic framework by utilizing both the inter-sequence base alignment probabilities and the intra-sequence base-pairing probabilities. The following subsections provide an overview of the proposed algorithm.

### Preliminary

To align *m* RNA sequences in a set **S** = {*s*_1_, ⋯ , *s_m_*}, we need to compute the following probabilities.

• *P_a_*(*x_i_* ~ *y_j_|***x**, **y**): For each pair sequence **x**, **y** ∈ **S**, *P_a_*( *x_i_* ~ *y_j_|***x**, **y**) is the probability that bases *x_i_* ∈ **x** and *y_j_* ∈ **y** are matched in the true (unknown) alignment. We can compute the posterior pairwise alignment probabilities using the pair hidden Markov model (PHMM) [40].

• *P_b_*(*x_i_* ~ *x_j_|***x**): For each sequence **x** ∈ **S**, *P_b_*( *x_i_* ~ *x_j_|***x**) is the probability that two bases *x_i_*, *x_j_* ∈ **x** form a base-pair. We can exploit different approaches, such as the McCaskill algorithm [41] or the CONTRAfold model [39], to compute the base-pairing probabilities.

We use these probabilities in the following probabilistic structural alignment scheme.

### Consistency transformation

Here, we use three types of probabilistic consistency transformations to modify the pairwise base alignment probabilities and base-pairing probabilities using the information from other sequences in the alignment. This modification makes these posterior probabilities suitable for constructing a consistent and accurate structural alignment.

#### Inter-sequence probabilistic consistency transformation for base alignment probabilities

In the first consistency transformation, we incorporate the information from other sequences in the alignment to improve the estimation of pairwise base alignment probabilities. The motivation of this transformation is that all the pairwise alignments induced from a given MSA should be consistent with each other. This means that if position *x_i_* (∈ **x**) aligns with position *z_k_* (∈ **z**) in the **x** – **z** alignment, and if *z_k_* aligns with position *y_j_* (∈ **y**) in the **z** – **y** alignment, then *x_i_* must align with *y_j_* in the **x** – **y** alignment. We can thus utilize the “intermediate” sequence **z** to improve the **x** – **y** alignment by making it consistent with the alignments **x** – **z** and **z** – **y**.

Based on this motivation, we introduced an enhanced probabilistic consistency transformation in PicXAA [34], which improves the original transformation proposed by Do *et al.*[*30*]. The enhanced transformation modifies the alignment probability for a base-pair *x_i_* ~ *y_j_*, by incorporating the alignment probability between *x_i_* and *z_k_* and that between *z_k_* and *y_j_.* This transformation can be written as:

where *P*(**x**◊**z**) represents the probability that **x** and **z** are homologous, defined as:

where ā is the optimal pairwise alignment of **x** and **z**.

This transformation improves the consistency of the **x** – **y** alignment with other pairwise alignments in the MSA, by incorporating information only from homologous sequences. In this way, we can obtain more probabilistically consistent estimate of the posterior alignment probabilities, which helps enhance the quality of the final MSA.

#### Intra-sequence probabilistic consistency transformation for base-pairing probabilities

In the second transformation, we incorporate the pairwise alignment information to the structural formation of the sequences. This transformation exploits this observation that the base-pairings in each sequence should be consistent with the pairwise base alignments induced from a given structural alignment. This means that if positions *y_j_* ~ *y_j_*_′_ form a base-pair in **y**, where *x_i_* (∈ **x**) aligns with *y_j_* (∈ **y**) and *x_i_*_′_, (∈ **x**) aligns with *y_j_*_′_ (∈ **y**), then *x_i_* ~ *x_i_*_′_ must form a base-pair in **x**. Thus, we can utilize the base alignment information to improve the estimation of the *x_i_* ~ *x_i_*_′_ base-pairing probability.

Based on this observation, Kiryu *et al.*[12] introduced a transformation for base-pairing probabilities, which was modified later in [42] as:

where *α* ∈ [0, 1] is a weight parameter between the target sequence **x** and rest of sequences. This transformation assumes that all sequences **y** ∈ **S** – {**x**} are homologous to the given sequence **x**. However, when we have a set of distantly related sequences in **S**, this assumption does not necessarily hold. To address this problem, here, we modify this transformation by improving the base-pairing probability using the information just from the closely related sequences to the given sequence **x**. Therefore, like the *inter-sequence consistency transformation*, we explicitly consider the relative significance of each sequence **y** ∈ **S** – {**x**} in improving the base-pairing probabilities in **x**.

Let **Z** = {**y** ∈ **S** – {**x**}*|***x** ◊ **y**} be the set of sequences in **S** – {**x**} that are homologous to **x**. The notation **x** ◊ **y** means **x** and **y** are homologous and functionally related to each other. Using only the relevant sequences, which are included in the set **Z**, we define this transformation as:

The second term in the right hand side of the above equation can be also written as:

using the identity function **I**{·}, where **I**{**x** ◊ **y**} = 1 if **y** is homologous to **x**, and **I**{**x** ◊ **y**} = 0 otherwise. In practice, we cannot judge with certainty whether two sequences are homologous or not. Thus, we describe this relationship probabilistically, using the expectation as: **E** [**I**{**x** ◊ **y**}] = *P*(**x** ◊ **y**), where *P*(**x** ◊ **y**) is the homology probability and can be estimated as described in the previous subsection. By replacing the identity functions with their expected values in the previous equation, we propose the following enhanced *intra-sequence probabilistic consistency transformation* as:

#### Probabilistic four-way consistency transformation for base alignment probabilities

In the third consistency transformation, we incorporate the structural information to the pairwise alignments. This transformation is based on the same observation that motivated the *intra-sequence consistency transformation;* that is, the pairwise base alignments induced from a given structural alignment should be consistent with the base-pairings in the corresponding pair sequence. However, this time, we utilize the base-pairing information to improve the **x** – **y** alignment.

Based on this motivation, Katoh and Toh introduced the four-way consistency transformation in [26] which was also latter implemented in [17]. We use this idea in a probabilistic fashion by incorporating the base alignment and the base-pairing probabilities as in [17]. This transformation is defined as:

where *β* ∈ [0, 1] is a weight parameter.

Using the sparsity of alignment and pairing probability matrices, we can efficiently implement these three transformations successively. The *inter-sequence consistency transformation* has a complexity of *O*(*µ*^2^*Lm*^3^), the *intra-sequence transformation* has a complexity of *O*(*µ*^3^*Lm*^2^), and *the four-way consistency transformation* has a computational complexity of *O*(*µ*^4^*Lm*^2^), where *µ* is the average number of non-zero elements per row (typically 1 ≤ *µ* ≤ 5 in real examples), *m* is the number of sequences, and *L* is the length of each sequence.

### Constructing the structural alignment

To find a valid structural alignment of a set of RNA sequences, we propose a two-step greedy approach that builds up the alignment starting from those regions with higher base-pairing and base alignment probabilities. The proposed greedy scheme extends the idea of PicXAA [34] to multiple RNA alignments. In PicXAA, we construct the multiple protein sequence alignment by successively inserting the most probable pairwise residue alignment into the final alignment. In the proposed algorithm, we add another step before the greedy graph construction step of PicXAA to better incorporate the secondary structure information in RNAs. This two-step alignment construction approach, along with *intra-sequence consistency transformation* and *four-way consistency transformation*, described in the previous subsection, helps PicXAA-R to effectively integrate both sequence and structural similarities to construct the final alignment. The proposed structural alignment approach is described in the following.

The greedy alignment approach we proposed in PicXAA [34] is conceptually similar to the one used in sequence annealing algorithms [29,35,36]. However, it should be noted that unlike sequence annealing, which greedily merges pairs of columns, we always add a single pairwise base alignment at a time, based on the consistency-transformed posterior alignment probabilities.

We represent the structural alignment as a directed acyclic graph ***G*** = (***V***, ***E***) where, ***V*** is the set of vertices and ***E*** is the set of directed edges. Each vertex *c*^(^*^i^*^)^ ∈ ***V*** corresponds to a column in the final alignment, and each directed edge *e* = (*c*^(^*^i^*^)^, *c*^(^*^j^*^)^) ∈ ***E*** implies that column *c*^(^*^i^*^)^ precedes column *c*^(^*^j^*^)^ in the given alignment. Each column *c*^(^*^i^*^)^ ∈ ***V*** consists of positions from different sequences that will appear in the same column in the final alignment.

When inserting a new pairwise base alignment, we should consider the following requirements to obtain a legitimate multiple RNA alignment:

• (Avoid Cycles) The alignment graph ***G*** should remain acyclic.

• (Left-Right Compatibility) In the first greedy step where we use structural information, we should consider left-right compatibility. That is, for any paired columns (*c, c*′), if column *c* appears in the left part of the stem in the final structure, then for each base *x_i_* ∈ *c* that pairs with some *x*_*i*′_ ∈ *c*′ of the same sequence **x**, we should have *i* <*i*′.

Thus, while we build up the alignment graph, we satisfy the structural constraints and alignment constraints by verifying whether the new inserted pairwise base alignment keeps the graph acyclic and left-right compatible.

The two-step alignment construction approach is as follows:

#### Step 1-Structural skeleton construction

In the first alignment construction step, we greedily insert the most probable alignments of base-pairs with high base-pairing probability. To this aim, we define the ordered set **B** as

**Figure 1:**
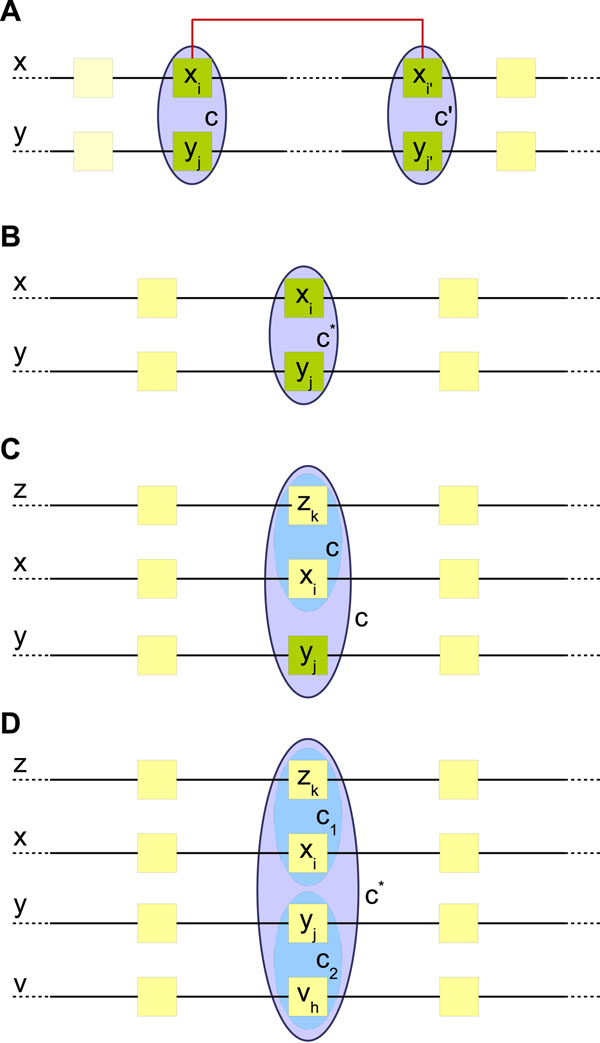
**Graph constructing process.** (A) Step 1-Structural skeleton construction: Adding a new base-pair (*x_i_*, *x*_*i*′_) and aligning that with its best match: (*y_j_*, *y*_*j*′_). (B-D) Step 2-Inserting highly probable local alignments: (B) Adding a new column (node) c*. (C) Extending an existing column (node) *c*. (D) Merging two columns (nodes) *c*_1_ and *c*_2_ into a single column (node) c*.

Here, **B** is the ordered set of base-pairs whose transformed base-pairing probability is larger than a threshold *T_b_* . The base-pairs in **B** are sorted in descending order according to their transformed base-pairing probability, . We successively pick the most confident base-pair (*x_i_*, *x_i_*_′_) from **B**. For a selected base-pair, we look for the best match among the members of **B**. That is, we seek for a pair (*y_j_*, *y_j_*_′_) ∈ **B** which belongs to another sequence **y** and satisfies the two compatibility conditions above in ***G*** while maximizing the following probability:

For this pair (*y_j_*, *y*_*j*′_), we insert two pairwise alignments (*x_i_* ~ *y_j_*) and (*x*_*i*′_ ~ *y*_*j*′_) into the alignment graph ***G****.* Figure 1A illustrates this process.

Upon inserting a new pair p* = (*x_i_*, *y_j_*) to ***G***, three scenarios may occur: (1) New column addition; (2) Extension of an existing column; or (3) Merging of two columns. The detailed description of the procedures needed for each case can be found in [34]. Later in this section, we provide a summary of those procedures. By successively inserting the most probable alignment for confident base-pairs, we construct the skeleton of the alignment enriched by structural information. Next, we complete this skeleton by greedily inserting highly probable base alignments.

#### Step 2-Inserting highly probable local alignments

In this step, we update the skeleton alignment obtained in the previous step by successively inserting the most probable pairwise base alignments into the multiple structural alignment, as in PicXAA [34]. Thus, we sort all remaining pairwise alignments (*x_i_*, *y_j_*) according to their transformed alignment probability  in an ordered set **A**. We greedily build up ***G*** by repeatedly picking the most probable pair in **A**, which is not processed yet, provided that it is compatible with the current alignment. Again, insertion of any pair p* = (*x_i_*, *y_j_*) to ***G*** will result in one of the scenarios of new column addition, extension of an existing column, or merging of two columns.

Here, we briefly discuss these three cases (For detailed description see [34]):

1. **New column addition:** We insert a new compatible vertex c* = {*x_i_*, *y_j_*} in ***G*** if neither *x_i_* nor *y_j_* belongs to some existing column in ***G****.* Figure 1B illustrates this process.

2. **Extending an existing column:** If only one of the bases in p*, let say *x_i_*, belongs to some vertex *c* ∈ ***V***, we should add the other base *y_j_* to the same vertex *c*. Figure 1C illustrates this process.

3. **Merging two vertices:** When *x_i_* ∈ *c*_1_ and *y_j_* ∈ *c*_2_ belong to two different vertices *c*_1_, *c*_2_ ∈ ***V***, we merge the vertices *c*_1_ and *c*_2_. Figure 1D illustrates this process.

After updating the graph as described above, we prune ***G*** to avoid redundant edges, thereby improving the computational efficiency of the construction process.

**Figure 2:**
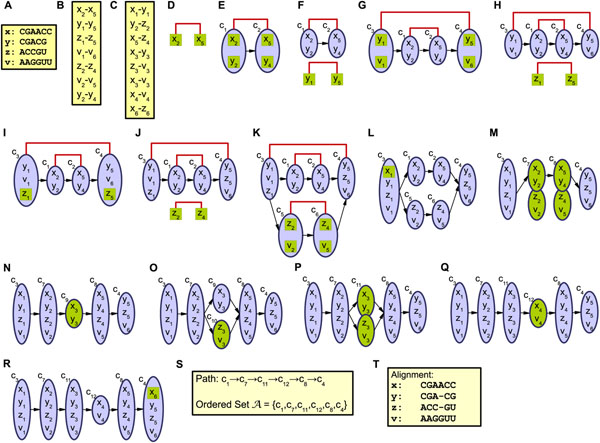
**An illustrative example for the graph construction process in PicXAA-R.** (A) The set of RNA sequences to be aligned. (B) The base-pairs are sorted according to their base-pairing probabilities. (C) The base alignments are sorted according to their transformed alignment probabilities. (D-K) Step 1- Structural skeleton construction: (D, E) Adding a new base-pair (*x*_2_, *x*_5_) and aligning that with its best match (*y*_2_, *y*_4_). (F, G) Adding a new base-pair (*y*_1_, *y*_5_) and aligning that with its best match (*v*_1_, *v*_6_). (H, I) Extending nodes *c*_3_ and *c*_4_ by adding the base-pair (*z*_1_, *z*_5_) to its best match (*y*_1_, *y*_5_). (J,K) Adding a new base-pair (*z*_2_, *z*_4_) and aligning that with its best match (*v*_2_, *v*_5_). (L-R) Step 2- Inserting highly probable local alignments: (L) Extending the node *c*_3_ by adding the base alignment (*x*_1_, *y*_1_). (M) Merging nodes *c*_1_ with *c*_5_ to include the base alignment (*y*_2_, *z*_2_) and merging nodes *c*_2_ with *c*_6_ to include the base alignment (*x*_5_, *z*_4_).(N) Adding a new node for the alignment (*x*_3_, *y*_3_). (O) Adding a new node for the alignment (*z*_3_, *v*_3_). (P) Merging nodes *c*_9_ and *c*_10_ to include the alignment (*x*_3_, *v*_3_). (Q) Adding a new node for the alignment (*x*_4_, *v*_4_). (R) Extending the node *c*_3_ by adding the base alignment (*x*_6_, *z*_6_). (S) The final alignment graph ***G***, which gives us the set ***A*** in a legitimate topological ordering. (T) The alignment obtained from ***A***.

Upon finishing the two-step graph construction, we use the obtained alignment graph ***G*** to find the multiple alignment. We use the depth-first search algorithm to order the vertices in ***V*** in an ordered set ***A*** = (*v*_1_, *v*_2_, ⋯ , *v_n_*) such that there is no path from *v_i_* to *v_j_* in ***G*** for any *i* > *j.* In the resulted ordered set ***A***, each member corresponds to a column in the alignment, and putting them together gives the alignment. Further details of the graph construction and alignment process can be found in [34]. An illustrative example for the graph construction process using PicXAA-R can be found in Figure 2.

### Discriminative refinement

As the final step, we apply a refinement step to improve the alignment quality in sequence regions with low alignment probability. We employ the iterative refinement strategy based on the discriminative-split-and-realignment technique that was introduced in PicXAA [34]. We repeat the following steps successively for each sequence **x** ∈ **S**:

1. Find **S_x_** ⊂ **S**, the set of similar sequences to **x** using the *k*-means clustering.

2. Align **x** with the profile of sequences in **S_x_**.

3. Perform the profile-profile alignment of  and **S** – **S_x_**.

This refinement strategy takes advantage of both the intra-family similarity as well as the inter-family similarity, thereby improving the alignment quality in low similarity regions without breaking the confidently aligned bases.

## Results and discussion

We use four different benchmark datasets: BRAliBase 2.1 [43], Murlet [12], BraliSub [44], and LocalExtR [44] to assess the performance of PicXAA-R on different alignment conditions. The first two are general datasets not specially designed for local RNA alignment testing while the last two datasets are designed to verify the alignment accuracy for locally similar RNAs.

We compared PicXAA-R with several well-known RNA sequence alignment algorithms:

ProbConsRNA 1.10 [30], MXSCARNA 2.1 [20], CentroidAlign [17], and MAFFT-xinsi 6.717 [26]. Among these techniques, ProbConsRNA uses only the sequence level information while the others take advantage of structural information. We picked these methods as they are among the fastest structural RNA aligners which yield high accuracy. There exists several other aligners such as RAF 1.00 [13], Murlet [12], Stemloc-AMA [29], LARA 1.3.2 [23], M-LocARNA [16], and R-Coffee [21], which have much higher complexity than MAFFT-xinsi (in some cases they are near 60 times slower) while their accuracy is usually worse or at least comparable to MAFFT-xinsi. Thus, the most complex algorithm that we compare our algorithm with will be the state-of-the-art technique, MAFFT-xinsi.

All the experiments have been performed on a 2.2GHz Intel Core2Duo system with 4GB memory. On all datasets we use two measurements to evaluate the performance of each alignment scheme: (1) *sum-of-pairs score* (SPS), which represents the percentage of correctly aligned bases; (2) *structure conservation index* (SCI) [45] that measures the degree of conservation of the consensus secondary structure for a multiple alignment. The SCI score is defined as  where *E_A_* is the minimum free energy of the consensus MSA as computed by RNAalifod [46] and Ē is the average minimum free energy of all single sequences in the alignment as computed by RNAfold [47].

On Murlet dataset, in addition to the SPS and SCI scores, we measure sensitivity SEN = TP*/*(TP + FN), Positive Predictive Value PPV = TP*/*(TP + FP), and Matthews correlation coefficient (MCC):

where true positive (TP) indicates the number of correctly predicted base-pairs, true negative (TN) is the number of base-pairs correctly predicted as unpaired, false negative (FN) is the number of not predicted true base-pairs, and false positive (FP) is the number of incorrectly predicted base-pairs.

In each table the total computational time for each algorithm is also reported in seconds.

Throughout the experiment we use the parameter setting of *α* = 0.4, *β* = 0.1, and *T_b_* = 0.5. These parameters are optimized manually using small datasets. Besides, we use McCaskill algorithm [41] to compute the base-pairing probabilities and RNAalifold [46] to find the induced consensus structure of the computed alignment.

### Results on BRAliBase 2.1

First, we evaluated the accuracy of PicXAA-R using the BRAliBase 2.1 alignment benchmark. Wilm *et al.*[43] has developed BRAliBase 2.1 based on hand-curated seed alignments of 36 RNA families taken from Rfam 7.0 database [48]. BRAliBase 2.1 contains in total 18,990 aligned sets of sequences each consists of 2, 3, 5, 7, 10, or 15 sequences (categorized into k2, k3, k5, k7, k10, and k15 reference sets) with average pairwise sequence identities ranging from 20% to 95%.

Table 1 summarizes the SPS and SCI scores along with the running time of each algorithm. As we see, MAFFT-xinsi has the highest average scores while it is two times slower than PicXAA-R. In comparison with other techniques PicXAA-R has similar scores which usually gets better as the number of sequences increases (k10 and k15).

**Table 1:** Performance evaluation on BRAliBase 2.1

Method	k2	k3	k5	k7	k10	k15	TIME
	SPS/SCI	SPS/SCI	SPS/SCI	SPS/SCI	SPS/SCI	SPS/SCI	
PicXAA-R	84.27 / 85.86	86.59 / 83.35	88.78 / 83.20	90.04 / 81.72	90.97 / 79.95	92.17 / 79.73	6502
ProbConsRNA	83.58 / 82.46	85.46 / 76.54	87.90 / 75.85	88.99 / 74.91	89.90 / 73.25	90.76 / 71.92	1444
MXSCARNA	85.02 / 90.67	86.57 / 85.56	88.43 / 83.44	89.40 / 80.89	90.17 / 78.34	91.26 / 77.18	6024
CentroidAlign	85.55 / 88.64	87.06 / 83.77	88.93 / 82.40	89.99 / 81.23	90.96 / 80.22	91.65 / 79.34	6443
MAFFT-xinsi	85.66 / 90.77	87.76 / 87.11	90.27 / 86.70	91.36 / 85.70	92.26 / 84.73	93.22 / 85.38	12386

**Figure 3:**
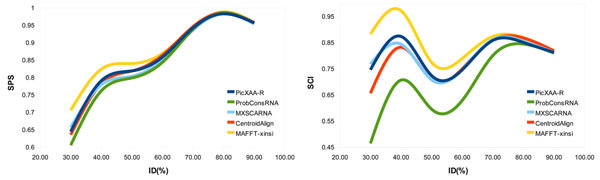
**Accuracy of alignment as a function of the average percent identity.** Comparing the accuracy in terms of SPS and SCI scores versus the average percent identity of the alignments in k5, k7, k10, and k15 reference sets of BRAliBase 2.1.

To more clearly compare these techniques, we provide the average SPS and SCI scores as a function of the average percent identity on k5, k7, k10, and k15 reference sets in Figure 3. As shown in this figure, for sequence identities less than 60% PicXAA-R outperform all the other schemes in terms of both scores except for MAFFT-xinsi which is two times slower than PicXAA-R. This observation shows that the proposed greedy approach can efficiently and effectively construct the alignment for low identity sequence sets. This was expected as in lower sequence identities the proposed greedy alignment construction approach can effectively detect local structural similarities.

### Results on BraliSub and LocExtR

The BraliBase 2.1 benchmark is not designed for local alignment testing and has reference alignments with just up to 15 sequences. Thus, Wang *et al.*[44] designed two types of datasets to verify the potential of RNA sequence aligners in dealing with local similarities in the alignment set: (1) BraliSub, the subsets of BraliBase 2.1 with high variability (containing 232 reference alignments); (2) LocalExtR, an extension of BraliBase 2.1 consisting total of 90 large-scale reference alignments categorized into k20, k40, k60, and k80 reference sets receptively with 20, 40, 60, and 80 sequences in each alignment.

Tables 2 and 3 summarize the performance measures on these datasets. As we can see, MAFFT-xinsi has the best accuracy but it is 2.5 times slower than PicXAA-R in BraliSub dataset and four times slower than PicXAA-R in LocExtR dataset. Besides, PicXAA-R outperforms MXSCARNA with average 6-7% in terms of SPS and SCI scores. It also outperforms CentroidAlign by average 1-2% in both scores.

**Table 2:** Performance evaluation on BraliSub

Method	k5	k7	k10	k15	TIME
	SPS/SCI	SPS/SCI	SPS/SCI	SPS/SCI	
PicXAA-R	73.90 / 51.39	75.06 / 42.37	74.02 / 35.75	75.43 / 31.29	101
ProbConsRNA	70.59 / 34.94	70.18 / 28.45	68.73 / 24.03	66.53 / 18.29	35
MXSCARNA	70.77 / 46.30	69.93 / 35.95	68.58 / 27.91	69.75 / 17.79	84
CentroidAlign	74.23 / 47.26	74.39 / 39.13	74.51 / 35.59	72.92 / 29.14	106
MAFFT-xinsi	78.28 / 57.60	78.56 / 52.10	78.48 / 44.75	79.23 / 38.79	261

**Table 3:** Performance evaluation on LocExtR

Method	k20	k40	k60	k80	TIME
	SPS/SCI	SPS/SCI	SPS/SCI	SPS/SCI	
PicXAA-R	71.46 / 17.43	77.52 / 16.08	80.19 / 11.00	82.51 / 10.73	999
ProbConsRNA	64.97 / 10.13	69.08 / 8.12	72.11 / 5.80	74.46 / 6.87	676
MXSCARNA	65.52 / 9.67	68.30 / 8.44	69.45 / 9.15	71.16 / 8.93	662
CentroidAlign	71.68 / 18.63	74.48 / 15.56	77.55 / 11.90	79.32 / 10.07	1359
MAFFT-xinsi	77.02 / 26.30	80.48 / 20.84	81.96 / 16.70	83.52 / 14.00	3791

These results confirm that PicXAA-R can efficiently yield an accurate structural alignment for a set of large number of locally similar RNAs.

### Results on Murlet dataset

Murlet dataset [12] consists of 85 alignments of 10 sequences obtained from the Rfam 7.0 database [48]. This dataset includes 17 families and there are five alignments for each family. The mean pairwise sequence identity varies from 40% to 94%. Table 4 shows the results on this dataset. We observe that PicXAA-R yields comparable accuracy with MAFFT-xinsi while PicXAA-R has much less complexity. In comparison with CentroidAlign, we have similar SPS and better SCI scores, while we are 3% better in terms of SEN score and 2% worse in terms of PPV score. However, for MCC score which compromises between sensitivity and specificity PicXAA-R outperforms CentroidAlign by 0.8%.

**Table 4:** Performance evaluation on Murlet dataset

Method	SPS	SCI	SEN	PPV	MCC	TIME
PicXAA-R	77.90	48.15	66.08	72.71	68.29	139
ProbConsRNA	76.26	37.47	56.79	78.12	65.10	40
MXSCARNA	74.67	44.28	64.06	74.58	68.37	120
CentroidAlign	77.99	47.80	63.08	74.88	67.48	146
MAFFT-xinsi	78.72	52.94	67.04	74.56	69.64	307

### Computational complexity analysis

**Figure 4:**
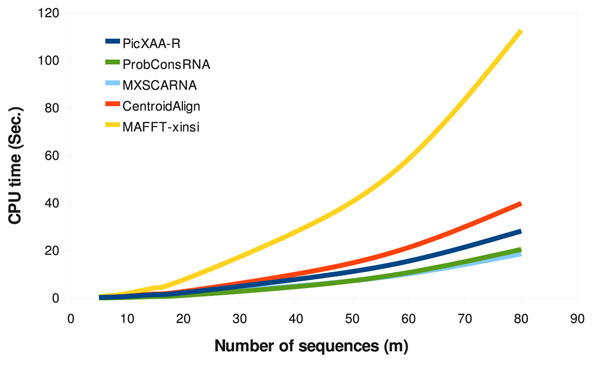
**Complexity analysis**. Comparing the dependency of different algorithms to the number of sequences in the alignment. The average running time are shown for sequences in BraliSub and LocExtR datasets.

Figure 4 shows the average CPU time for different algorithms as a function of the number of sequences in the alignments in BraliSub and LocExtR datasets. As we see, the complexity of MAFFT-xinsi grows much faster than other algorithms as the number of sequences increases, while the complexity of PicXAA-R smoothly grows with number of sequences. We also see that PicXAA-R stands between MXSCARNA and CentroidAlign in terms of CPU time. However, as shown in the previous subsections, we outperform both these techniques in datasets consisting sequences with local similarity and low pairwise identity.

## Conclusions

In this paper, we proposed PicXAA-R, a probabilistic structural RNA alignment technique based on a greedy algorithm. Using a set of probabilistic consistency transformations, including a novel *intra-sequence consistency transformation*, we incorporate the folding and alignment information of all sequences to enhance both the posterior base-pairing and base alignment probabilities. We utilize these enhanced probabilities as the building blocks of the two-step greedy scheme which builds up the alignment starting from sequence regions with high local similarity and high base-pairing probability. As shown in several experiments, PicXAA-R can efficiently yield highly accurate structural alignment of ncRNAs. This performance is more vivid for datasets consisting sequences with local similarities and low pairwise identities. To the best of our knowledge, PicXAA-R is the fastest structural alignment algorithm after MXSCARNA among all the current RNA aligners while it significantly outperforms MXSCARNA on local datasets like BraliSub and LocExtR. High speed implementation of PicXAA-R as well as its accuracy makes it a practical tool for structural alignment of large number of ncRNAs with low sequence identity which is very helpful for novel ncRNA prediction.

## Authors' contributions

Conceived the algorithm: SMES, BJY. Implemented the algorithm and performed the experiments: SMES. Analyzed the results: SMES, BJY. Wrote the paper: SMES, BJY.

## Competing interests

The authors declare that they have no competing interests.
